# Influence of cultural values and hierarchical social norms on buying counterfeits online: a 17-country study

**DOI:** 10.3389/fpsyg.2024.1394660

**Published:** 2024-07-19

**Authors:** Anastasia Kononova, Patricia Huddleston, Moldir Moldagaliyeva, Heijin Lee, Saleem Alhabash

**Affiliations:** ^1^Department of Advertising and Public Relations, College of Communication Arts and Sciences, Michigan State University, East Lansing, MI, United States; ^2^Center for Anti-Counterfeiting and Product Protection (A-CAPP), Michigan State University, East Lansing, MI, United States

**Keywords:** counterfeit purchase, cultural dimensions, hierarchical social norms, injunctive norms, online shopping, individualism/collectivism, power distance

## Abstract

**Introduction:**

As a globally prevalent phenomenon, buying counterfeit products harms consumers, economies, societies, governments, and the environment. The study examined the hierarchy of injunctive normative influence (personal vs. societal) on counterfeit purchase intentions and trends in non-deceptive (known) counterfeit purchase behavior. The current research expands the hierarchical norms approach by examining how the cultural values of power distance and individualism–collectivism predict injunctive normative perceptions and counterfeit buying intention and behavior.

**Methods:**

A cross-sectional survey (N = 13,053) of consumers from 17 nations, administered in seven languages, explored cross-country differences in perceived social norms about buying counterfeits.

**Results:**

The findings of multilevel moderated mediation analyses showed that personal injunctive norms (perceived acceptance of buying counterfeits by close friends) mediated the relationship between societal injunctive norms (perceived acceptance for buying counterfeits by peers in the same country) and the outcome variables. Selected paths of the mediation model were moderated by the two cultural dimensions.

**Discussion:**

Theoretical implications are discussed within the context of cultural orientations’ and social norms’ roles in informing risky behavior, and practically, within the context of awareness-raising and behavior-change interventions.

## 1 Introduction

Purchasing counterfeit products poses significant dangers to consumers, economies, governments, and the environment worldwide (Grossman and Shapiro, 1988; Ferrell and Hartline, 2011; Wilson and Kinghorn, 2016; OECD and EUIPO, 2021). The Lanham Act defines trademark counterfeiting as the “reproduction, counterfeit, copy, or colorable imitation of a registered mark,” which is applied to “labels, signs, prints, packages, wrappers, receptacles or advertisements” and “such use is likely to cause confusion, or to cause mistake, or to deceive” [Lanham Act, 15 U.S.C. § 1,114(1)]. Though legal definitions vary by country and within countries, along with the prevalence of counterfeit supply and demand, the most common attribute of counterfeit goods is that these goods or their packaging have an unauthorized or spurious trademark. The counterfeit market, spanning nearly all product categories, ballooned from $449 billion in 2021 (OECD and EUIPO, 2022) to $3 trillion in 2022 due to the prevalence of e-commerce, especially during the COVID-19 pandemic (Handfield, 2021).

Using or consuming counterfeit products may injure consumers due to poor product quality, lack of safe ingredients, and lack of safe and regulated manufacturing (United Nations Office on Drugs and Crime, 2013). For example, the surge in substandard, falsified, and counterfeit (SFC) medications is particularly troublesome. An estimated 10% of all medications sold in middle- and lower-income countries and 50% of those sold online are SFC (World Health Organization, 2017). The Pharmaceutical Security Institute (PSI, 2023) documented 6,615 global incidents of pharmaceutical seizures in 2022 (half recorded in North America alone), a 10% increase from 2021 and a 50% increase from 2018 (4,405 incidents). Recent reports indicate that overdose deaths involving counterfeit drugs increased from 2 to 4.7% from 2019 to 2021 (O’Donnell et al., 2023). Further, about 250,000 children die annually because of SFC medications (Yadav and Rawal, 2015; World Health Organization, 2017; Sample, 2019).

While the direct harms of using SFC medications to consumers are clear, counterfeiting in other product categories also poses significant health and safety risks. American Apparel and Footwear Association (2022) study tested 47 counterfeit “clothing, footwear, and other accessories” and found that over a third of these products “failed to comply with U.S. product safety standards,” as they “contained dangerous levels of arsenic, cadmium, phthalates, lead, and more that have been shown to cause adverse health outcomes.” Counterfeiting of airbags, auto and airplane parts, food, and medical equipment has also become increasingly prevalent. Recently, Sarah Loughran, 34 years old, died in a fatal car crash, and upon inspection, it was determined that the airbag in her car was counterfeit (Merwin, 2023).

Consumers are often deceived into buying counterfeits, thinking the product is authentic, but they also often buy fakes knowingly (non-deceptively). In the current study, non-deceptive counterfeit purchase is conceptualized as a planned behavior. We compare predictors of two behavioral outcomes: intentions to buy fake products in the future and past non-deceptive counterfeit purchasing.

There is a growing need to better understand the social and psychological mechanisms at play when purchasing and intending to purchase counterfeits. Though individually motivated, counterfeit purchase is influenced by social perceptions of counterfeit purchase prevalence and acceptance. Furthermore, such social influence may vary across cultures. The current study leverages a 17-country cross-sectional survey to examine the hierarchical nature of perceived social norms. The study extends the hierarchical norms approach by focusing on injunctive, rather than descriptive norms, whereby societal injunctive norms’ (e.g., among peers in the same country) influence on counterfeit purchase behavior is mediated by personal injunctive norms (e.g., among close friends). Given that counterfeit buying is a global phenomenon that affects many countries, we investigate how this hierarchical norms relationship (Patrick et al., 2012) is moderated by country-level cultural values of power distance and individualism–collectivism (Hofstede, 1979; Hofstede and Bond, 1984, 1988; Hofstede et al., 2010). We considered the cultural attributes of power distance and individualism–collectivism to be directly relevant to social influences and counterfeit buying because the former deals with the importance of social relationships and the latter concerns perceptions of authority and status.

The study’s significant contribution lies first in examining the hierarchical norms hypothesis within the context of buying counterfeits. Second, we investigate the relationship between cultural dimensions and social norms of buying counterfeits using multilevel analyses. Finally, unlike past research focusing on a unidimensional aspect of counterfeit purchase, our study examines – in parallel – counterfeit purchase intentions and non-deceptive counterfeit purchase.

## 2 Literature review

### 2.1 Buying counterfeits: intention and behavior

In the current study, we examine two behavioral outcomes: counterfeit purchase intention and non-deceptive counterfeit purchase. Purchasing products and services is a planned behavior in most instances (Bian and Veloutsou, 2007). This definition is especially relevant to studying counterfeit buying in which consumers engage knowingly. In the current study, we define such behavior as a non-deceptive counterfeit purchase (NDCP) and operationalize it by asking participants to report past instances of buying counterfeits knowing they were not authentic. Counterfeit purchase intentions (CPI) refer to the perceived probability that consumers will buy counterfeit products in the future.

While the plan to behave in a certain way does not always materialize, it is correlated with the actual behavior. Past research applying theories of reasoned action and planned behavior suggests that behavioral intention predicts behavior and, furthermore, reinforces future intentions and behaviors. In the context of our study, we suggest that counterfeit product purchase intention is associated with actual purchase of fakes (e.g., Gentry et al., 2006; Bian and Veloutsou, 2007; Kim and Karpova, 2010; Cheng et al., 2011; Chiu and Leng, 2015; Patiro and Sihombing, 2016; Garas et al., 2023). The hypotheses in the current study are informed by this positive relationship, thus, they have the same directionality for both behavioral outcomes. Yet, we use the two measures separately to grasp the differences between planned and actual behavior. As purchase behavior is intertwined with social norms, the present study examines injunctive norms - personal and societal - as predictors of the two behavioral outcomes.

### 2.2 The social norms of buying counterfeits

Social norms provide an understanding of acceptable vs. unacceptable behaviors in a society. They are defined as “rules and standards that are understood by members of a group and that guide and/or constrain social behavior without the force of laws” (Cialdini and Trost, 1998, p. 152). As inherently social beings, humans influence and are influenced by others’ behavior through social interaction (Rimal and Lapinski, 2015; Myers and Twenge, 2019). Normative influence occurs when individuals feel the pressure to conform to social norms (Yanovitzky and Rimal, 2006), which are negotiated and constructed within the group and serve as “standards for the individual’s perception and judgment” (Sherif and Sherif, 1953, p. 202). Humans adjust their attitudes and behaviors based on normative perceptions to fit in a reference group within their social environment.

There are two types of social norms, descriptive and injunctive (Cialdini et al., 1991, p. 203), also defined as subjective norms (Manning, 2009). Descriptive norms are based on perceptions of “what most people do,” and injunctive norms refer to individual’s perceptions of “what most people approve or disapprove of” or what people should do. In contrast to descriptive norms that reflect beliefs of behavioral prevalence, injunctive norms often carry moral judgments by highlighting the anticipated social value of one’s actions, where unfavorable actions are socially sanctioned and favorable ones enhance perceived group belongingness (Chung and Rimal, 2016). Injunctive norms can be further classified as personal and societal injunctive norms. Personal injunctive norms refer to behavior expected by close reference groups such as friends and family. Societal injunctive norms refer to behavior expected by distant reference groups such as peers in the same town or city (Melnyk et al., 2022; Yang, 2018). While we have provided an overview of descriptive norms for understanding injunctive norms, the focus of this paper is on the hierarchical influence of injunctive personal and societal norms on past purchases and purchase intention to purchase counterfeits.

The Theory of Normative Social Behavior [TNSB; see Chung and Rimal (2016), for a review] proposes that injunctive norms moderate the relationship between descriptive norms and behavioral outcomes, which are sensitive to behavioral, individual, and contextual factors. When descriptive and injunctive norms are aligned, this has the strongest positive influence on environmental intentions (Smith et al., 2012). It was found that descriptive and injunctive norms equally influence behavioral intention (Manning, 2009); however, descriptive norms are stronger in predicting consumer behavior than injunctive norms (Manning, 2009; Melnyk et al., 2021). The act of buying counterfeits cannot be understood exclusively from a consumer behavior perspective because it is qualitatively different than, for example, buying a new t-shirt or pack of gum. It entails elements of risk and potential for social disapproval. Such risks extend beyond legal risks to health and safety risks. As for social approval or disapproval, consuming to conspicuous trademarks (e.g., a luxury bag with a prominent logo) might be sensitive to social costs to one’s image. The focus on injunctive social norms in this study stems from an understanding that they “are universally viewed as one of the most important factors in human social life” (Gavrilets, 2020, p. 14). While descriptive norms reflect perceptions of behavioral prevalence (i.e., how many people are enacting the behavior), injunctive norms reflect perceptual expectations of how one should behave that stem for notions of social acceptance of that behavior, thus positioning injunctive norms as self-fulfilling prophecies for behaving in a particular way (Gavrilets, 2020).

The literature on counterfeit-related topics suggests that social acceptance and peer influence are key determinants of counterfeit purchase behaviors (Faria, 2013; Thaichon and Quach, 2016; Bhatia, 2018; Moon et al., 2018; Abdullah and Yu, 2019; Singh et al., 2021). In cultures and communities where buying fakes is common (descriptive norms) and accepted (injunctive norms), social norms increase the likelihood of counterfeit purchasing (Kastanakis and Balabanis, 2012, 2014; Swoboda et al., 2014). When injunctive normative influences are weakened, and individuals prioritize integrity and lawfulness, then consumers avoid counterfeit purchases due to ethical considerations, value consciousness, and personal gratification (e.g., Ang et al., 2001; Wang et al., 2005; Francis et al., 2015; Singh et al., 2021). In a study of Indian consumers, attitudes toward counterfeit behavior mediated the relationship between luxury counterfeit purchase injunctive personal (subjective) norms and purchase intentions (Singh et al., 2021). However, a study of Generation Y consumers found no relationship between injunctive personal norms and counterfeit consumption (Francis et al., 2015), which the authors attributed to the “rebellious attitude of counterfeit owners” (p. 597). Overall, these findings provide a blueprint for the potential of group and cultural variability to interfere with how norms impact counterfeit purchase behavior.

In the current study, we propose that the proliferation of e-commerce and social commerce facilitated the prevalence of counterfeit goods; thus, on a global level, consumers are aware of the availability of counterfeits and the potential purchase by their peers. Counterfeit prevalence is magnified when average users and influencers promote fake products to their audiences. For example, Amazon sued two influencers in 2020 for promoting counterfeit products through third-party sellers (Palmer, 2020). Nike also recently filed a lawsuit against Nicholar Tuinberg and Eben “Cedaz” Fox for promoting and reviewing fake sneakers (Falk, 2023). Anecdotally, users, influencers, and illicit sellers are flooding social media to promote, rate, and rank counterfeits. It is safe to surmise that knowledge of this behavior is commonplace in the digital environment. Accordingly, we predict that examining injunctive norms and variability in perceptions of the acceptance of buying counterfeits might be more influential in explaining counterfeit purchase behaviors. Such influences are context-dependent (Smith et al., 2012; Khan et al., 2023) and vary in the reference groups to which one attributes the normative influence.

### 2.3 The hierarchical norms of buying counterfeits

Social norms explain behavior and can be used to change it, especially when interventions adjust misconceptions about behavioral prevalence and acceptance within a group. The Social Norms Approach (SNA) (Berkowitz, 2005) capitalizes on the norm-behavior relationship by enhancing behavioral compliance. It achieves this by contrasting one’s own perceptions about the prevalence (descriptive norms) and acceptance (injunctive norms) of the behavior to the actual prevalence and acceptance of the behavior within one’s reference group. Variability in normative perceptions of reference groups impacts the strength of norm-behavior association. The closeness of reference groups significantly influences both perceived descriptive and injunctive norms. Perceived norms within close (proximal) reference groups, like family and close friends (personal norms), influence counterfeit behavior more strongly than perceived norms about distal reference groups, like peers in the same town/city or country (societal norms) (Larimer et al., 2009; Yang, 2018; Melnyk et al., 2021).

Previous literature examining the impact of normative influences on consumer behavior demonstrates that injunctive norms are particularly dependent on how close the reference groups are (Cho, 2016; Yang, 2018). For example, Cho (2016) found that personal injunctive norms were strongly correlated with individuals’ alcohol consumption behavior, while the distal reference group’s approval was not related to the outcome. Moreover, Yang (2018) extended these findings by showing that expected approval from close friends positively predicted alcohol consumption; however, approval from distal peers negatively predicted this behavior. It was reasoned that people may disagree with less important groups to emphasize their identification with more significant referents (Yang, 2018). Furthermore, the strength and immediacy of a source were shown to profoundly affect its societal influence (Latané, 1981). Sources that are both proximate and immediate are more likely to shape behaviors, primarily because conforming to such norms usually garners social approval, enhancing an individual’s likability and social status (Geber, 2019; Geber and Hefner, 2019). In line with these findings, as people are more likely to identify with proximal groups and perceive their behaviors as more pronounced (Chung and Rimal, 2016), proximal relationships exert a greater influence on consumer behaviors than those with more distal relationships. Consequently, relationships with these closer groups substantially influence consumer behaviors more than those with more distant associations.

Patrick et al. (2012) suggested that normative influences on behavior are ordered hierarchically. Specifically, personal norms are embedded within societal norms, and collectively, they hierarchically influence behavior. Patrick et al. (2012) found that personal norms mediated the relationship between societal norms and alcohol consumption around one’s 21st birthday in the United States. While focusing on the difference between proximal and distant normative influences, the authors did not make a clear distinction between descriptive and injunctive norms, where subpopulation norms (societal, distant) were operationalized as descriptive and proximal norms included measures of both perceived approval (injunctive) and perceived behavior within close social circles (Patrick et al., 2012). Alhabash et al. (2021), on the contrary, focused on descriptive social norms, where descriptive personal norms mediated descriptive societal norms’ association with self-reported celebration drinking on Halloween. This relationship was also predicted by social media behaviors of posting and interacting with alcohol-related posts. A meta-analysis confirmed that social norms referencing a close (vs. abstract) group member are more effective (Melnyk et al., 2022). Therefore, the current study’s focus on injunctive norms extends the hierarchical norms approach.

**Figure 1:**
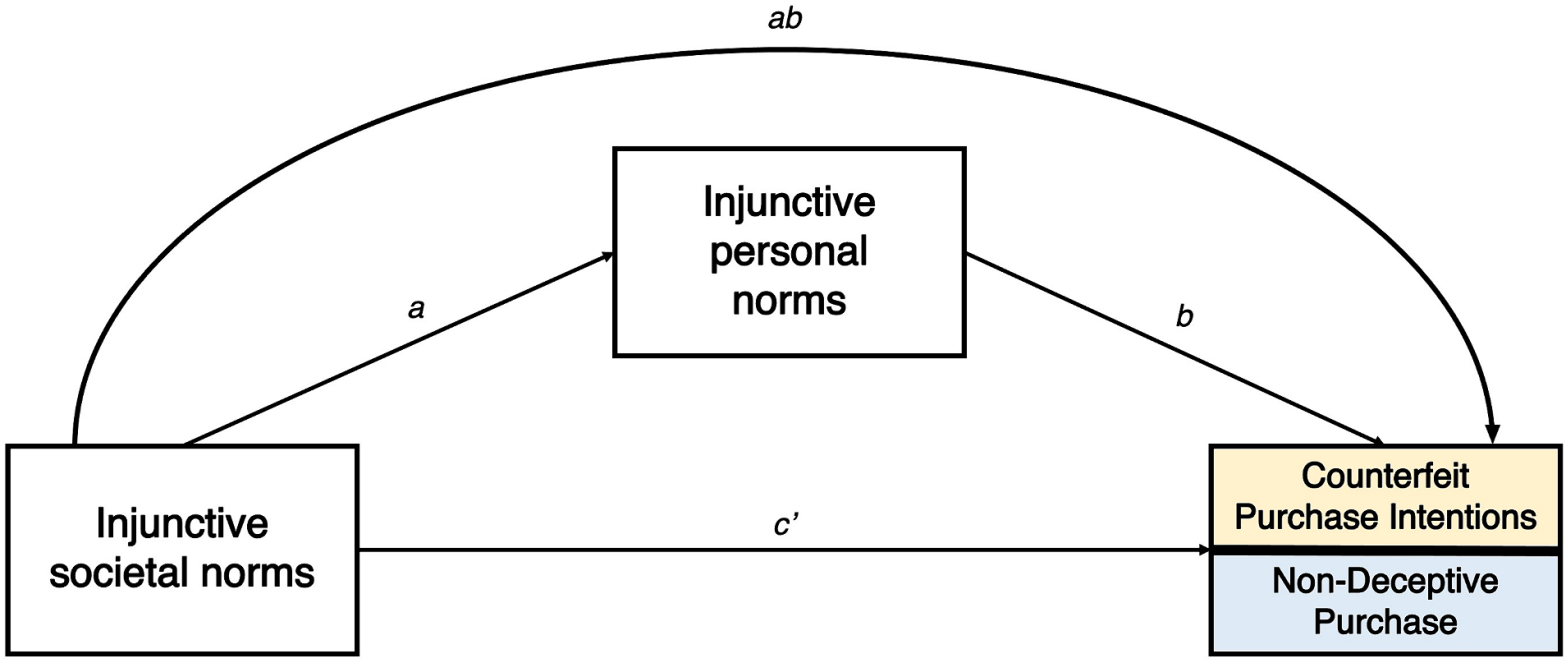
Model depicting the mediation effect of injunctive personal norms on the relationship between injunctive personal norms and counterfeit-related behavioral variables.

The current study applies the hierarchical norms hypothesis to personal and societal injunctive normative influences to extend the existing literature that supports the hierarchical order for social and descriptive norms. However, as mentioned previously, the focus of this paper is on personal and societal injunctive norms, not descriptive norms. Based on the empirical evidence reviewed, we propose that perceptions of distant others’ acceptance of buying counterfeits might have a weaker association with individuals’ behavior than the association between perceived acceptance of this behavior among close (proximal) friends and family members. Developing this argument further, we argue that perceived acceptance among proximal others (personal injunctive norms) strengthens the relationship between societal injunctive norms (perceived approval of distant others) and counterfeit purchase intention and behaviors. Thus, we state the following hypotheses (see Figure 1).

H1: Personal injunctive norms positively mediate the relationship between societal injunctive norms and counterfeit purchase intentions (CPI).

H2: Personal injunctive norms positively mediate the relationship between societal injunctive norms and non-deceptive counterfeit purchase (NDCP).

### 2.4 Cultural values and social norms

#### 2.4.1 Hofstede’s cultural values

Social norms and cultural values are highly interconnected (Liu and Lapinski, 2024). The cultural orientation of a group (e.g., national group, cultural subgroup) implies prevalent and subtle expressions of social normative attitudes and behaviors (Lapinski et al., 2007; Gelfand et al., 2011; Saracevic et al., 2022). National culture can serve as the most common denominator for a country’s citizens, including their sub-group membership, and distinguish countries based on collective attitudes, values, beliefs, and behaviors. This common denominator emphasizes the importance of examining these processes at the individual and group (e.g., national) levels (Schwartz, 1999, 2006).

Extensive cross-cultural research has identified values and psychological dimensions that sketch a country’s national and cultural identity (Vinken et al., 2004; Maleki and De Jong, 2013). Research on national cultural orientations pinpoints cultural dimensions as national attributes that express cultural values, attitudes, and behaviors that distinguish cultural and national groups (Hofstede, 1979; Hofstede and Bond, 1984). Cultural dimensions “summarize the extent to which cultural groups are found empirically to differ from one another in terms of psychological attributes such as values, beliefs, self-construals, personality, and behaviors” (Smith and Bond, 2020, p. 971). Cultural orientation encompasses “normative cognitive (thoughts about life and the universe), conative or directional (inclination toward or selection of a particular course of action), and affective (what is felt as important and desirable) elements” (Carter, 1991, p. 165). At the group level, value orientations manifest themselves through aggregated “perceptions, thoughts, norms, attitudes, feelings, and motivational inclinations,” which translate into shared normative behaviors (Carter, 1991, p. 165). Such orientations and values become central to one’s group belonging. Cultural, normative, perceptual, and behavioral commonalities within a culture drive everyday behavior and represent common group-level inclinations, though not devoid of intra-cultural variability (Schwartz, 2006).

Hofstede’s (1979) original work factor analyzed survey data from 40 countries to identify four major cultural dimensions: power distance (PD), individualism–collectivism (IDV), uncertainty avoidance (UA), and masculinity-femininity (MAS), which later was relabeled into “motivation towards achievement and success” (Hofstede, 2023). Later studies proposed two additional dimensions: long- vs. short-term orientation (LTO) (Hofstede and Bond, 1988) and indulgence vs. restraint (IND) (Hofstede et al., 2010).

IDV, is a bipolar dimension, where cultures vary between being individualistic, “where people are supposed to look after themselves and their families,” and collectivistic. It is reflected by people’s belonging to in-groups and collectivities where they are supposed to look after others in exchange for loyalty (Hofstede and Bond, 1984, p. 419). PD deals with “the extent to which less powerful members of institutions and organizations accept that power is distributed unequally.” In high PD cultures, people show a greater respect for authority and social status, compared with low OD cultures. UA, which reflects a society’s approach to coping with the unpredictability of the future (Hofstede, 2023), refers to “the extent to which people feel threatened by ambiguous situations and have created beliefs and institutions that try to avoid” uncertain situations (Hofstede and Bond, 1984, p. 419). MAS relates to a culture’s orientation and motivation for achievement and success. A high MAS score reflects the focus on competition, achievement, and success. In contrast, a low MAS score indicates the priority for caring for others, where success is seen in terms of life quality (Hofstede, 2023). LTO evaluates whether societies favor preserving traditional values (low score) or adapting pragmatically with a focus on education and thrift to prepare for the future (high score) (Hofstede, 2023). The IND dimension is “the extent to which people try to control their desires and impulses, based on how they were raised” (Hofstede, 2023).

Hofstede’s cultural values have been linked to norm violations. For example, a 57-country study found that PD positively correlated with normative violation scenarios of physical confrontation, verbal confrontation, and social ostracism, yet the correlation was negative for gossip and non-action. Furthermore, more individualistic participants were less likely to endorse physical confrontation, verbal confrontation, and social ostracism, yet favored gossip and non-action (Eriksson et al., 2021). While this latter study did not directly measure perceived social norms, it provides insights into how cultural orientations and values influence perceptions of social relations and behaviors. Research on the relationship between cultural dimensions (measured at the national and individual levels) and perceived social norms to predict behavior and behavioral compliance is evolving. Pei et al.’s (2020) three studies during the COVID-19 pandemic showed a strong positive relationship between collectivistic cultural orientation and perceived injunctive norms related to behavioral compliance with COVID-19 guidelines and protective health behaviors. Moreover, countries deemed more collectivistic “demonstrated lower growth rate in both COVID-19 confirmed cases and deaths,” where normative perceptions, which are sensitive to collectivistic cultural inclination, resulted in higher injunctive normative perceptions (e.g., most people’s approving of social distancing), which in turn resulted in higher behavioral compliance, and ultimately, lower COVID-19 cases (both prevalence and death) (Pei et al., 2020).

Hofstede’s research routinely linked cultural dimensions (at the national level) to consumer behavior (de Mooij and Hofstede, 2011). For example, past evidence suggests an inverse relationship between PD and impulse buying (Zhang et al., 2010) and a positive relationship between IDV and impulse buying (Kacen and Lee, 2002) and IDV and green consciousness (Kim, 2011). High PD consumers prefer mass-market (vs. niche) brands (Wang et al., 2022) and have less price sensitivity for purchasing products or services (Lee et al., 2020). Similarly, consumers with individualistic values often make decisions autonomously, ignoring their social groups’ views largely because they are not accustomed to collective decision-making processes (Wagner, 1995).

#### 2.4.2 The case for power distance and individualism/collectivism

Though all of Hofstede’s cultural dimensions correlate with aspects of consumer behavior, the current study primarily focuses on PD and IDV for their potential strong relationship with normative perceptions. Unlike the other four dimensions, these two reflect attitudes toward social groups and have been primarily understood through people’s self-perception in social dynamics, known as self-construal. These dimensions make people more susceptible to others’ approval when they perceive themselves as interdependent with others (vs. independent) (Markus and Kitayama, 2014; Cohen et al., 2021; Park et al., 2023). For example, individuals from collectivist backgrounds select brands that strengthen their ties to their group, thereby enhancing their collective identity (Luna and Gupta, 2001). In contrast, those from individualistic societies prefer brands that highlight their uniqueness, thus distinguishing themselves from others. As expressions of a nation’s orientation toward group conformity, or lack thereof, IDV, we suggest, is parallel to normative perceptions and their influence on behavior.

On the other hand, PD has been shown to reflect how consumption is intertwined with expressions of social hierarchy (e.g., Kim and Zhang, 2011; Jiang et al., 2021). PD correlates with the consumption of luxury brands, explained by the need to enhance and express one’s social status and social approval (Kim and Zhang, 2011; Bharti et al., 2021; Bizarrias et al., 2023). Therefore, we anticipate that PD will significantly correlate with personal and societal injunctive norms and counterfeit purchase behaviors and intentions.

Cultural values’ association with counterfeit purchases is stronger than economic factors such as income or price perceptions (Franses and Lede, 2015). Past consumer behavior research documented that cultural values and perceived social norms influence behavioral tendencies and consumption patterns. Below, we review studies investigating the relationship between PD and IDV and consumer attitudes and behaviors toward counterfeits. The current study focuses on PD and IDV as indices of national cultural orientation, which includes indices of the overall country’s cultural orientation (group level) rather than at the individual level.

##### 2.4.2.1 Power distance

Findings linking power distance (PD) to consumption patterns, including the purchase of counterfeit goods, are mixed. For example, in countries with lower PD — where society tends to be less accepting of unequal power distribution — there is a higher tendency for counterfeit product presence (Santos and Ribeiro, 2006). However, Jiang et al. (2021) observed that people with low power distance beliefs, who typically downplay societal hierarchies, prefer less conspicuous luxury items, choosing them for their value expression rather than as status symbols. Countries with higher PD were found to have higher levels of piracy than more egalitarian societies (Ronkainen and Guerrero-Cusumano, 2001), and conspicuous consumption and flaunting of wealth are shown to be more tolerated in high-PD cultures (Wang et al., 2022). This juxtaposition of findings indicates that cultural beliefs about social status and perceived power distribution can influence consumer behavior in diverse ways. Furthermore, research on general and specialized patterns of consumer behavior, like buying luxury goods, showed significant correlations between PD – both as an individual-level and country-level factor – in predicting the purchase of luxury products in general and specific types of luxury products luxury products (e.g., Ronkainen and Guerrero-Cusumano, 2001; Santos and Ribeiro, 2006; Jiang et al., 2021; Wang et al., 2022).

Given that high PD countries accept differential social powers and hierarchies, individuals in those countries are keen on climbing the social ladder; thus, their consumption is leveraged to signal social status (actual and desired), and they are more inclined to buy counterfeit luxury products when financial resources are limited. Santos and Ribeiro’s (2006) findings suggest the reverse could also be true. Consumers in countries with low PD orientation might care about social hierarchy and status less and, thus, are willing to endure social costs to buy counterfeits, leading to higher purchases of fakes.

Based on the literature reviewed above, we predict that the mediation model proposed in H1 (Figure 1) will vary across countries with different PD scores. Specifically, we posit the following.

H3: The relationships between injunctive societal norms, injunctive personal norms, and CPI (a-, b-, and c’-paths) will differ based on the power distance cultural attribute of a country.

H4: The relationships between injunctive societal norms, injunctive personal norms, and NDCP (a-, b-, and c’-paths) will differ based on the power distance cultural attribute of a country.

##### 2.4.2.2 Individualism–collectivism

The culture-norm-behavior relationship is best articulated within the context of cultural orientations in a country or among sub-group members that influence the intensity of normative perceptions to enhance cooperative normative beliefs and behaviors. In collectivistic cultures (e.g., Mexico), the social value of a brand was found to be higher and more influential than in individualistic countries (e.g., United States). Moreover, the relationship between social norms and purchase intention was negative in the individualistic sample (i.e., United States) and positive in the collectivistic sample (i.e., Mexico) (Yang, 2014).

Individualism–collectivism (IDV) correlates with consumer behaviors (e.g., Kacen and Lee, 2002) and explains variability in counterfeit purchase perceived social norms and behavioral intentions (e.g., Faria, 2013; Swoboda et al., 2014). Studies investigating the relationship between IDV demonstrate more consistent results than those focusing on power distance. For example, highly individualistic countries have less software piracy (Marron and Steel, 2000; Ronkainen and Guerrero-Cusumano, 2001), and the positive effect of subjective norms on counterfeit purchase intentions is strengthened in collectivist countries (Phau and Teah, 2009; Swoboda et al., 2014). Normative susceptibility, i.e., making purchase decisions based on expectations of what would impress others, was positively related to Chinese consumers’ perceptions of counterfeits and purchase intention; however, collectivism was not related to perceptions and purchase intention of counterfeits (Wang et al., 2005; Phau and Teah, 2009). Consumers from highly collectivist cultures are more influenced by social norms, perceiving buying counterfeits as socially acceptable, thus expressing greater purchase intentions. In contrast, those from individualistic cultures prioritize individual personal ethics and thus express lower purchase intentions (Sharma et al., 2022). However, the influence of IDV might be transitory. Teah et al. (2015) found that the relationship between collectivism and counterfeit attitudes was positive and significant among mainland Chinese consumers, but not their Taiwanese counterparts. Teah et al. (2015) explained these findings by arguing that Taiwanese consumers are moving away from traditional, collectivistic values.

While several studies pinpointed a relationship between cultural values, social norms, and counterfeit behavior, Melnyk et al.’s (2022) meta-analysis of the effect of social norms on consumer behavior found no relationship between the interaction of individualism–collectivism, uncertainty avoidance, and social approval of target behaviors. However, this study did not include counterfeit purchase behavior in their analysis. Only a few studies investigated the relationship between cultural values, social norms, and counterfeit purchase behavior. The associations between counterfeit buying, injunctive personal subjective norms, and attitudes were positive and significant only among participants from China, a collectivistic culture, but not in more individualistic Canada (Faria, 2013). In a three-country comparative study of Chinese, Romanian, and German consumers, Swoboda et al. (2014) found that the relationship between counterfeit buying subjective norms and intentions was positive in all samples; yet, the association was stronger among Romanian and Chinese consumers (more collectivistic), respectively, compared to German consumers (more individualistic). Additionally, DiRienzo and Das (2020) found that people in individualistic countries were more structurally capable of confronting illicit trade than people in collectivistic nations. Collectivistic countries were suggested to lack the structural capacity to confront risks of illicit trade, including sales of counterfeit products.

Based on the existing empirical evidence reviewed above, we hypothesized the following.

H5: The relationships between injunctive societal norms, injunctive personal norms, and CPI (a-, b-, and c’-paths) will differ based on the IDV cultural attribute of a country, where higher collectivism is associated with higher injunctive norms and CPI.

H6: The relationships between injunctive societal norms, injunctive personal norms, and NDCP (a-, b-, and c’-paths) will differ based on the IDV cultural attribute of a country, where higher collectivism is associated with higher injunctive norms and NDCP.

## 3 Materials and methods

### 3.1 Survey design and participants

The current study looks at how PD and IDV cultural dimensions moderate the relationship between injunctive societal and personal norms on counterfeit-related behavioral outcomes.

This study used an online global cross-sectional survey of consumers from 17 countries (*N* = 13,053). Participants were recruited from Argentina (*n* = 769), Australia (*n* = 760), Brazil (*n* = 769), Canada (*n* = 770), China (*n* = 776), Egypt (*n* = 761), India (*n* = 770), Italy (*n* = 769), Kenya (*n* = 770), Mexico (*n* = 781), Nigeria (*n* = 765), Peru (*n* = 756), South Korea (*n* = 771), Spain (*n* = 760), United Arab Emirates (*n* = 770), United Kingdom (*n* = 766), and the United States (*n* = 770). The countries were selected to represent six of the seven continents (except Antarctica). We also selected countries known for high counterfeit seizures, e.g., U.S., Brazil, Nigeria (OECD and EUIPO, 2022) and high sources of counterfeit product origin (e.g., China, India). Countries with low counterfeit trade (e.g., Australia, South Korea) were also included in our sample for greater variability (Global Organized Crime Index, 2023).

The survey was administered in Arabic, Chinese, English, Italian, Korean, Portuguese, and Spanish (two versions were used for Spain and Spanish-speaking Latin American countries). Non-English versions of the survey were translated and back translated to English by a professional translation firm and were validated by a panel of industry experts who are native speakers of each language. In each country, we used three even-split sampling quotas for gender, generational groups, and income levels (lower, middle, upper).

Participants’ gender was evenly split: females (49.64%), males (49.63%), and “Other” (0.73%). The mean age of the sample was 38.46 years old (*SD* = 14.05, *Range* = 18 to 85). Most participants were employed (65.29%), college-educated (attended college or attained an associate/bachelor’s degree; 58.57%), married (63.11%), with at least one child living with them (63.64%). Participants reported that three others lived with them in the same household (*M* = 2.99, *SD* = 1.71). Most participants (97.92%) have shopped online at least once in the past 12 months.

### 3.2 Survey procedure

The study was determined exempt by the Institutional Review Board (IRB) at Michigan State University in the United States due to the anonymous and low-risk nature of participation. The survey was administered through www.Qualtrics.com, and participants were recruited by Qualtrics Panels through partnering with country-based third-party vendors. The English version of the survey was designed on Qualtrics. Upon back-translation and validation of different translations, we used the “Languages” function on Qualtrics to input question translations to other languages. Using metadata, the survey administration language was programmed to correspond with each participant’s country where they took the survey, however, participants could choose any language from a drop-down menu to accommodate multilingual countries. Upon recruitment, participants read and indicated approval of the informed consent form and agreed to the General Data Protection Regulation [GDPR] guidelines statement regarding the protection of their personal data. To ensure meeting quotas, participants reported demographic information in the beginning of the questionnaire. Participants were asked to indicate the frequency of their counterfeit purchase. Relevant to this study, participants evaluated items corresponding with societal and personal injunctive norms followed by items for counterfeit purchase intentions. Participants were provided with incentives in accordance with the panel vendor in each country. Upon data collection, Qualtrics Panels assessed data quality and eliminated unreliable responses (with replacement to reach sampling quotas per country). On average, participants completed the survey in about 19 min (this manuscript is part of a larger study).

### 3.3 Measures

The current study used multilevel analysis with survey measures as level-1 (individual) variables. Country and publicly available country averages for cultural dimensions were used as level-2 (group) predictors (Hofstede, 2023) (see Table 1).

**Table 1:** Power distance and individualism/collectivism scores for countries included in the study (Hofstede, 2023).

**Country**	**Power distance**	**Individualism/collectivism**
Argentina	49	51
Australia	38	73
Brazil	69	38
Canada	39	72
China	80	43
Egypt	80	13
India	77	24
Italy	50	53
Kenya	70	4
Mexico	81	34
Nigeria	80	0
Peru	64	20
South Korea	60	58
Spain	57	67
United Arab Emirates	74	36
United Kingdom	35	76
United States of America	40	60

#### 3.3.1 Injunctive social norms measures

We used three items (Park and Smith, 2007) to measure perceived injunctive societal and personal norms: (1) “approve of me buying counterfeit products;” (2) “endorse my buying of counterfeit products;” and (3) “would support that I buy counterfeit products.” The reference group for societal norms was “peers in the same country,” and that for personal norms was “close friends.” Items were rated on a seven-point Likert-type scale anchored by “Strongly Disagree” and “Strongly Agree.” Upon satisfactory factor and reliability analyses, items were averaged per participant for societal injunctive norms (*Eigenvalue* = 2.65, *% of* Var*iance Explained* = 88.25%, *Cronbach’s α* = 0.933) and personal injunctive norms (*Eigenvalue* = 2.76, *% of Variance Explained* = 91.88%, *Cronbach’s α* = 0.956).

#### 3.3.2 Counterfeit purchase intentions

Counterfeit purchase intentions (CPI) were measured using three seven-point Likert-type items (Park and Smith, 2007): “I intend to buy counterfeit products,” “I will likely buy counterfeit products,” and “It is possible that I will buy counterfeit products.” Upon satisfactory factor analysis (*Eigenvalue* = 2.61, *% of Variance Explained* = 86.96%) and reliability analysis (*Cronbach’s α* = 0.925), the three items were averaged per participant.

We performed multi-group CFA in R with the study data to ensure measurement invariance at several levels. We used three latent variables in the model, Injunctive Social Norms (3 items), Injunctive Personal Norms (3 items), and Counterfeit Purchase Intention (CPI) (3 items). The results of testing for configural, metric, and scalar invariance indicated that the model did not significantly change at each level of constraint. CFI indices changed by less than 0.01; and TLI, RMSEA, and SRMS values were within acceptable ranges at each level (Appendix 1).

Country-level scalar validity and reliability for injunctive societal norms, injunctive personal norms, and CPI were satisfactory (see Appendix 2).

#### 3.3.3 Non-deceptive counterfeit purchase

Participants indicated the frequency of non-deceptive counterfeit purchase (NDCP) within the past 12 months using seven-category ordinal scales ranging from “Never” to “Daily or almost daily.”

### 3.4 Data analysis

To test hypotheses, we performed multilevel modeling, using MLmed computational macro for SPSS (Rockwood, 2019). This software allows testing for the relationships between variables at different levels (e.g., properties of individuals vs. properties of countries). Because we conducted the survey in multiple countries, we expected that the variance in dependent measures would be explained not only by individual-level (within-group) factors, such as individual responses, but also country-level (between-group) predictors derived from attributes of each nation. This means that both respondents’ perceptions of counterfeit and related phenomena and their belonging to certain groups (e.g., nation states) will account for changes in the dependent variables (DVs). In our analysis, country was used as a cluster, 2-level variable to test if country (group) means and individual averages would be significantly correlated with the DVs. Other 2-level variables represented two cultural attributes: power distance (PD) and individualism–collectivism (IDV). The independent and dependent variables included in the model were 1-level variables. Injunctive societal norms was the main predictor, and counterfeit purchase intention and non-deceptive counterfeit purchase were entered in the model as criterion variables.

The advantage of the MLmed macro is that it makes it possible to perform multilevel mediation, moderation, and conditional analyses. Specifically, it allows up to three individual-level mediators and up to three country-level moderators (one for *a path*, one for *b path*, and one for *c’ path*), along with 1- and 2-level covariates. In the proposed model, in accordance with the hierarchical norms framework (Patrick et al., 2012), we used injunctive personal norms as a 1-level mediator.

IDV and PD were included as 2-level moderators. The limitation of the MLmed macro is that it allows testing for the conditional effects of a moderator on only one path at a time. Thus, we ran three models to test for the moderation of the direct effects of (1) injunctive societal norms on a DV (*c’ path*), (2) injunctive societal norms on injunctive personal norms (*a path*), and (3) injunctive personal norms on a DV (*b path*).

In summary, we ran 12 models using MLmed macro to test for the conditional effects of two 2-level moderators (PD and IDV) on paths a, b, and c’ (one model per path) with two DVs (CPI and NDCP) entered in models one at a time. Injunctive societal norms and injunctive personal norms were used in all models as an IV and mediator, respectively. Three 1-level covariates: age, gender, and religiosity were included in all analyses as the strongest and significant demographic predictors of each DV.

## 4 Results

### 4.1 Hierarchical norms

Hypotheses 1 and 2 predicted that personal injunctive norms would positively mediate the relationship between societal injunctive norms, on one hand, and CPI and NDCP, respectively, on the other.

#### 4.1.1 Path a (injunctive societal norms → injunctive personal norms)

**Figure 2:**
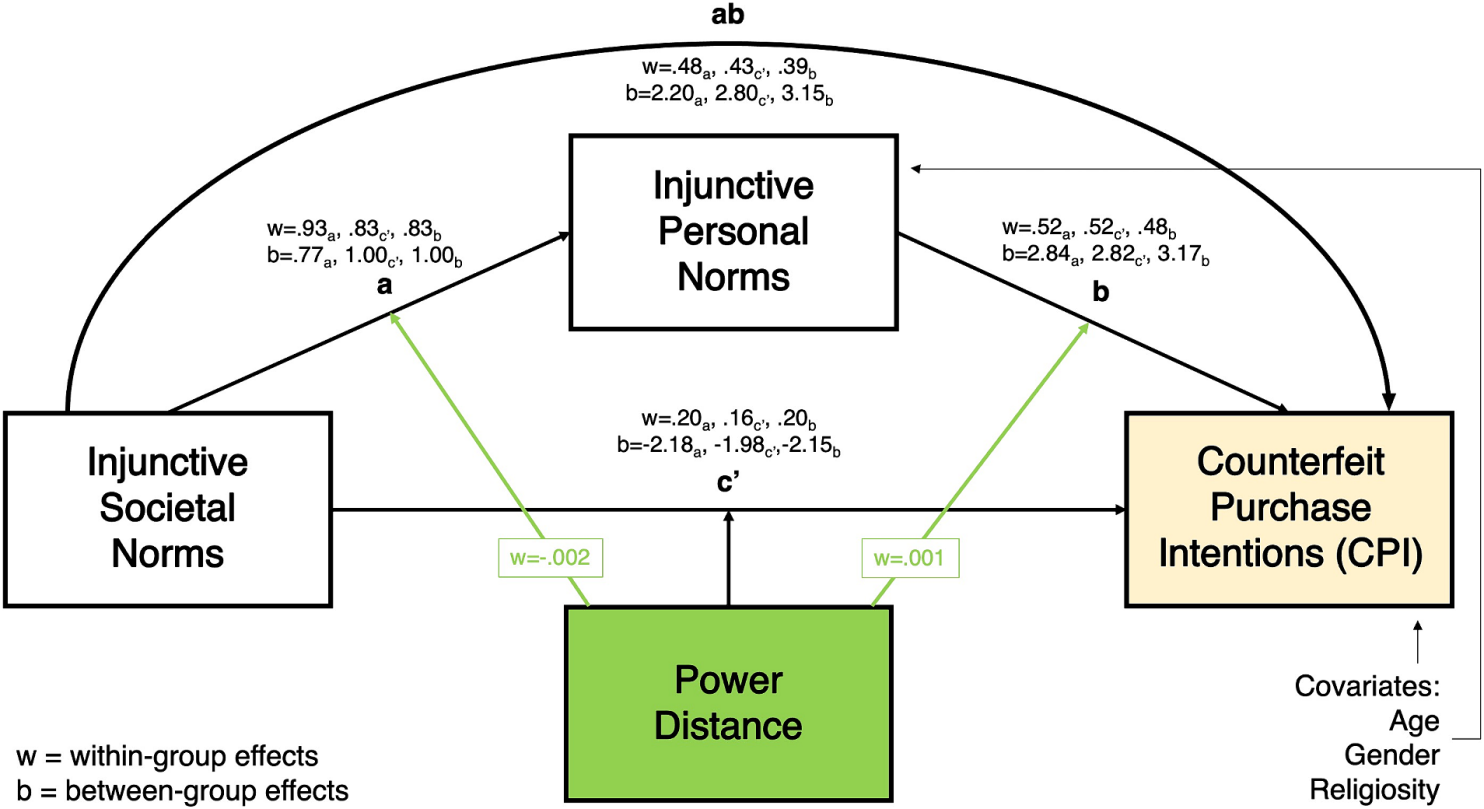
Power distance moderating the relationship between injunctive societal norms and counterfeit purchase intentions, mediated by injunctive personal norms.

**Figure 3:**
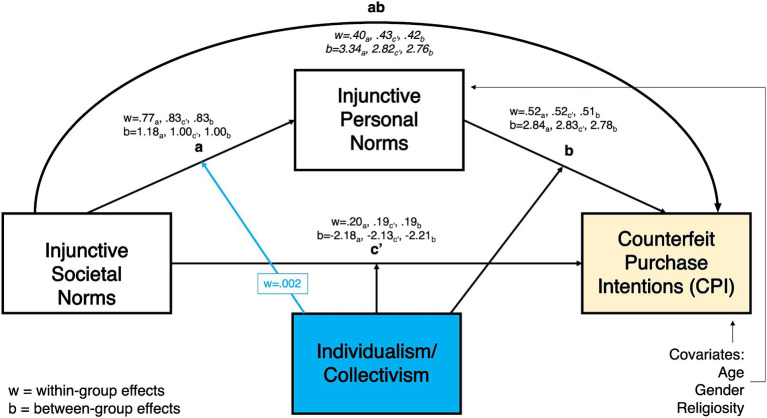
Individualism/collectivism moderating the relationship between injunctive societal norms and counterfeit purchase intentions, mediated by injunctive personal norms.

**Figure 4:**
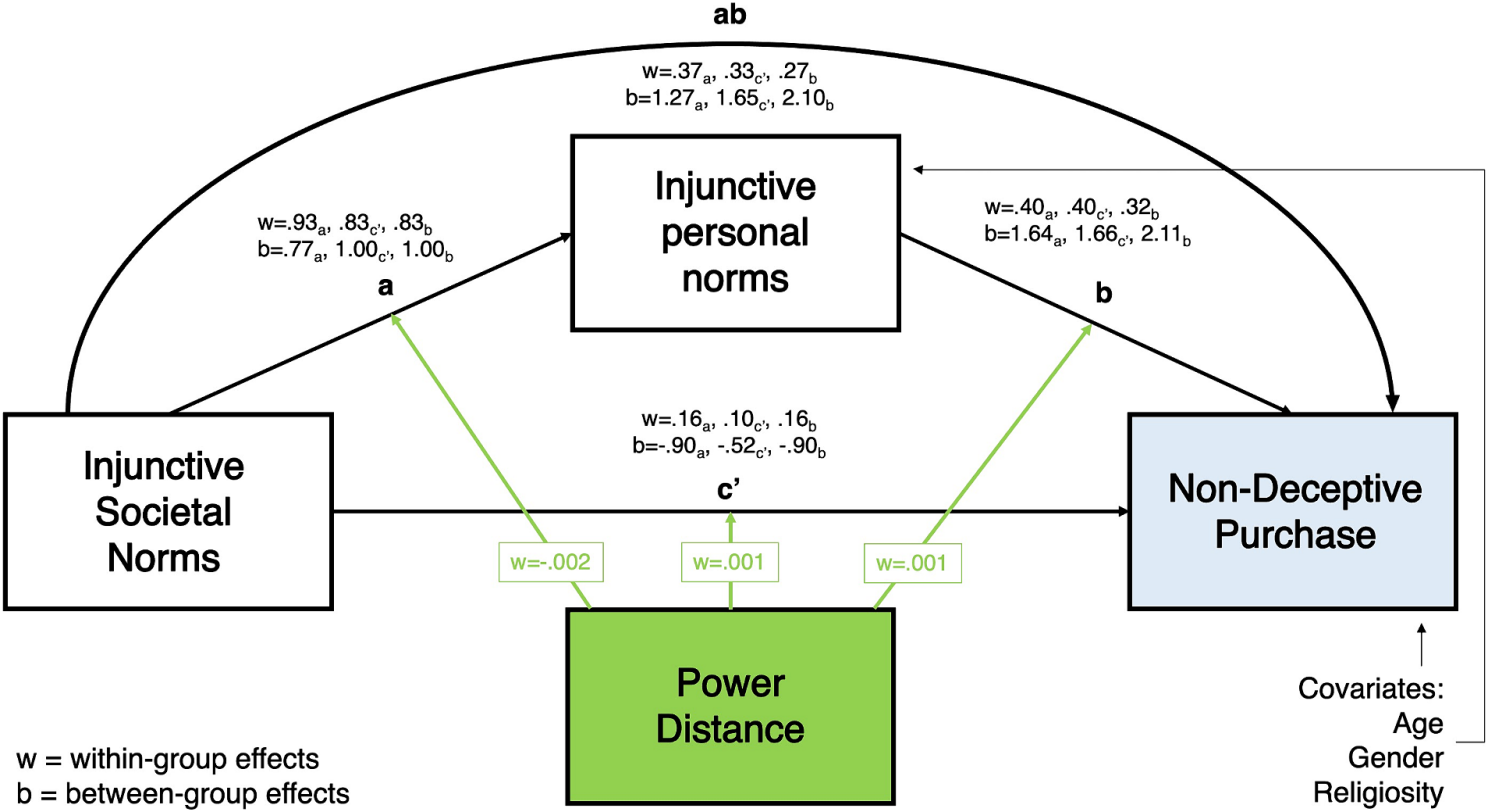
Power distance moderating the relationship between injunctive societal norms and non-deceptive counterfeit purchase, mediated by injunctive personal norms.

**Figure 5:**
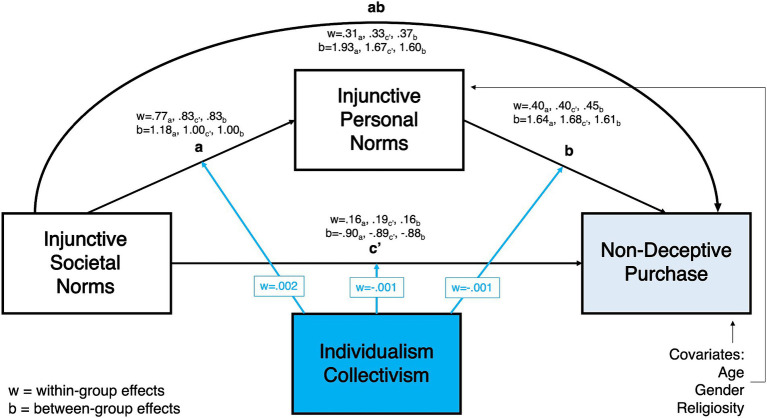
Individualism/collectivism moderating the relationship between injunctive societal norms and non-deceptive counterfeit purchase, mediated by injunctive personal norms.

As shown in Figures 2–5, all moderated mediation multilevel models consistently showed injunctive societal norms as a significant positive predictor of injunctive personal norms at within-group levels (CPI: wth. Coeff. = 0.765–0.929, LL = 0.746–0.889, UL = 0.787–0.968; NDCP: wth. Coeff. = 0.767–0.928, LL = 0.746–0.889, UL = 0.787–0.968). An increase in the total sample’s belief and country-specific sample averages related to the acceptance of counterfeit buying behavior in the society was associated with the increase in the belief about the acceptance of this behavior in close social circles.

#### 4.1.2 Path b (injunctive personal norms → CPI/NDCP)

Path-b positive relationships between injunctive personal norms and each DV were significant in all models, as well (CPI: wth. Coeff. = 0.511–0.518, LL = 0.483–0.498, UL = 0.538–0.539; NDCP: wth. Coeff. = 0.323–0.448, LL = 0.272–0.418, UL = 0.374–0.478). The more respondents believed that their close friends and family approved of buying counterfeit goods, the higher their reported CPI and NDCP were.

#### 4.1.3 Path c’ (injunctive societal norms → CPI/NDCP; direct effect)

Within-group direct relationship between injunctive societal norms and the two DVs in all models were significant. In the total sample, belief about the acceptance of counterfeit buying by distant peers (societal norms) was positively associated with the CPI and NDCP (CPI: wth. Coeff. = 0.192–0.196 LL = 0.165–0.174, UL = 0.215-0.220; NDCP: wth. Coeff. = 0.098–0.192, LL = 0.046–0.162, UL = 0.151–0.222, Figures 2–5). Interestingly, injunctive societal norms negatively predicted CPI at the between-group level, meaning that as the country group means for societal norms increased, the country’s average CPI decreased (CPI: btw. Coeff. = −2.132- -2.211, LL = –3.573 – –3.095, UL = –1.254 – –0.694 Figures 2, 3). In other words, countries’ aggregated beliefs about the acceptance of counterfeit buying in society translated into lower country-average intentions to buy counterfeits. Such significant and negative between-subjects effects repeated in the models with IDV as a moderator for NDCP (btw. Coeff. = −0.894 – –0.884, LL = –1.817 – –1.563, UL = –0.259 – –0.205, Figure 5). However, they were not significant in the models with power distance as a moderator (Figure 4).

#### 4.1.4 Path ab (injunctive societal norms → CPI/NDCP; indirect effect through injunctive personal norms)

Within-group indirect effects in all models with PD as a moderator were significant (CPI: coeff. = 0.393–0.482, LL = 0.353–0.454, UL = 0.430–0.509, Figure 2; NDCP: coeff. = 0.266–0.372, LL = 0.224–0.347, UL = 0.307–0.397, Figure 4), where injunctive personal norms mediated the relationship between injunctive societal norms and CPI. The tests of within-group indirect effects in the models with IDV as a moderator revealed similar significant mediation results (CPI: coeff. = 0.297–0.428, LL = 0.379–0.441, UL = 0.415–0.445, Figure 3; NDCP: coeff. = 0.307–0.370, LL = 0.288–0.348, UL = 0.325–0.395, Figure 5). These findings suggest that injunctive societal norms are strengthened by injunctive personal norms when explaining variance in the two DVs. H1 and H2 were supported.

To examine the associations between injunctive norms and the two DVs, we ran an additional mediation model in R (lavaan 0.6.17 package) with both CPI and NDCP included in the model at the same time. Both outcome variables were highly correlated (coef. = 0.87, SE = 0.07, *p* < 0.001). All model paths were found to be significant (*p* = <0.005), with b, ab, and c paths producing higher coefficients for CPI than NDCP (b-path coeff. = 0.57_CPI_ vs. 0.45_NDCP_; ab = path coef. = 0.48_CPI_ vs. 0.38_NDCP_; c-total effect coef. = 0.66_CPI_ vs. 0.55_NDCP_). The direct effect of injunctive societal norms on the two DVs was almost identical (c’-path coeff. = 0.179_CPI_ vs. 0.176_NDC_), suggesting that it is the injunctive personal norms that drove the predictive differences between behavioral intention and past behavior outcomes.

### 4.2 PD as a 2-level moderator

Hypotheses 3 and 4 stated that the relationships between injunctive societal norms, injunctive personal norms, and CPI (H3) and NDCP (H4) (a-, b-, and c’-paths) would differ based on the power distance cultural attribute of a country.

First, we present model fit findings for PD (Table 2) moderation models. For each group of models, between- and within-group variance percent values were obtained by calculating intraclass coefficients (ICC). Specifically, ICC was derived by dividing random effects estimates by the total estimates of random effects and level-1 residuals. Second, we present findings pertaining to the study’s hypotheses and research questions.

**Table 2:** Individual (Level-1 Residual) and group (Random effects) estimates for the model with power distance as a moderator and **(A)** counterfeit purchase intentions and **(B)** non-deceptive purchase as DVs.

**Criterion variable**	**Moderated path**	**(A)**			**(B)**		
		**Counterfeit purchase intentions**			**Non-deceptive purchase**		
		**Coeff. (SE)**	**Wald *Z***	**CI**_**LL, UL**_	**Coeff. (SE)**	**Wald *Z***	**CI**_**LL, UL**_
Level-1 Residual Estimates							
Injunctive Personal Norms as a DV	c’ model	1.04 (0.01)	78.20 ***	1.01, 1.07	1.04 (0.01)	78.20 ***	1.01, 1.07
	a model	1.04 (0.01)	78.20 ***	1.01, 1.06	1.04 (0.01)	78.20 ***	1.01, 1.06
	b model	1.04 (0.01)	78.20 ***	1.01, 1.07	1.04 (0.01)	78.20 ***	1.01, 1.07
Behavioral Outcome as a DV	c’ model	1.33 (0.02)	78.20 ***	1.30, 1.37	1.57 (0.02)	78.18 ***	1.53, 1.61
	a model	1.33 (0.02)	78.20 ***	1.30, 1.36	1.57 (0.02)	78.18 ***	1.53, 1.61
	b model	1.33 (0.02)	78.20 ***	1.30, 1.36	1.57 (0.02)	78.18 ***	1.53, 1.61
Random Effect Estimates							
Injunctive Personal Norms as a DV	c’ model	0.02 (0.01)	1.32 *	0.01, 0.06	0.02 (0.01)	2.32 *	0.01, 0.06
	a model	0.03 (0.01)	2.13 *	0.01, 0.07	0.03 (0.01)	2.13 *	0.01, 0.07
	b model	0.02 (0.01)	2.32 *	0.01, 0.06	0.02 (0.01)	2.32 *	0.01, 0.06
Behavioral Outcome as a DV	c’ model	0.06 (0.03)	2.06 *	0.02, 0.15	0.02 (0.01)	1.95 †	0.01, 0.07
	a model	0.05 (0.02)	2.26 *	0.02, 0.11	0.02 (0.01)	2.14 *	0.01, 0.05
	b model	0.06 (0.03)	2.05 *	0.02, 0.14	0.02 (0.01)	1.95 †	0.01, 0.06

c’ path, injunctive societal norms → behavioral outcome; a, injunctive societal norms → injunctive personal norms; b, injunctive personal norms → behavioral outcome; †*p* < 0.10, *p < 0.05, **p < 0.01, ***p < 0.001.

The PD moderation models with CPI as a DV indicated an overall good fit for variables at both levels predicting injunctive personal norms and counterfeit purchase intention (level-1 Wald *Z*s = 78.2; level-2 Wald *Z*s = 2.06–2.32). About 2.4% of the variance in injunctive personal norms and 3.8% of variance in intention was explained by country (Table 2A). The PD moderation models with NDCP indicated good fits at both levels (level-1 Wald *Z*s = 78.2; level-2 Wald *Z*s = 1.95–2.32). About 2.4% of the variance in injunctive personal norms and 1.4% of variance in non-deceptive purchase was explained by country (Table 2B).

The within-group conditional effect of PD on the association between injunctive societal and personal norms (a-path) was significant and negative (wth coeff. = −0.002, LL/UL = –0.002- –0.001). In high PD countries, the link between societal and personal injunctive norms was weaker than in low PD countries (Figures 2, 4).

The within-group conditional effect of PD on the relationship between injunctive personal norms and CPI (b-path) was also significant (wth coeff. = 0.001, LL/UL = 0.0001–0.001). In countries with high PD index, greater injunctive personal norms were positively associated with increased CPI (Figure 2, significant path moderation is highlighted by green arrows). Thus, H3 was partially supported by indicating the significant PD moderation of paths a and b with CPI as a DV.

All conditional effects of PD were significant in the models with NDCP as the DV. As reported above, PD negatively moderated the relationship between injunctive societal and personal norms. The relationships between injunctive societal norms and NDCP (c’ = path) (wth coeff. = 0.001, LL/UL = 0.0002–0.002) as well as injunctive personal norms and NDCP (b-path) (wth coeff. = 0.001, LL/UL = 0.001–0.002) were stronger in high than low PD countries (Figure 4, significant path moderation is highlighted by green arrows). H4 was supported with PD moderating paths a, b, and c’ with NDCP as a DV.

No direct between-group relationship between PD and injunctive personal norms and CPI and NDCP were significant. Additional mixed-models analysis also indicated no significant relationships between PD and injunctive societal norms.

### 4.3 IDV as a 2-level moderator

Hypotheses 5 and 6 stated that the relationships between injunctive societal norms, injunctive personal norms, and CPI (H5) and NDCP (H6) (a-, b-, and c’-paths) would differ based on the individualism–collectivism cultural attribute of a country.

The IDV moderation models with CPI as a DV indicated an overall good fit for variables at both levels predicting injunctive personal norms and intention (level-1 Wald Zs = 78.2; level-2 Wald *Z*s = 2.10–2.32). About 2.5% of the variance in injunctive personal norms and 4% of the variance in CPI were attributed to country (see Table 3A). The IDV moderation models with NDCP as a DV. Good model fits were found at both levels predicting injunctive personal norms and non-deceptive purchase (level-1 Wald *Z*s = 78.2; level-2 Wald *Z*s = 1.93–2.32). About 2.4% of the variance in injunctive personal norms and 1.4% of variance in NDCP was explained by country (see Table 3B).

**Table 3:** Individual (Level-1 Residual) and group (Random effects) estimates for the model with individualism/collectivism as a moderator and **(A)** counterfeit purchase intentions and **(B)** non-deceptive purchase as DVs.

Criterion variable	**Moderated path**	**(A)**			**(B)**		
		**Counterfeit purchase intentions**			**Non-deceptive purchase**		
		**Coeff. (SE)**	**Wald *Z***	**CI**_**LL,**_ **UL**	**Coeff. (SE)**	**Wald *Z***	**CI**_**LL,**_ **UL**
Level-1 residual estimates							
Injunctive personal norms as a DV	c’ model	1.03 (0.01)	78.20 ***	1.01, 1.07	1.03 (0.01)	78.20 ***	1.01, 1.07
	a model	1.04 (0.01)	78.20 ***	1.01, 1.06	1.04 (0.01)	78.20 ***	1.01, 1.06
	b model	1.04 (0.01)	78.20 ***	1.01, 1.07	1.04 (0.01)	78.20 ***	1.01, 1.07
Behavioral outcome as a DV	c’ model	1.33 (0.02)	78.20 ***	1.30, 1.36	1.57 (0.02)	78.18 ***	1.53, 1.61
	a model	1.33 (0.02)	78.20 ***	1.30, 1.36	1.57 (0.02)	78.18 ***	1.53, 1.61
	b model	1.33 (0.02)	78.20 ***	1.30, 1.36	1.57 (0.02)	78.18 ***	1.53, 1.61
Random effect estimates							
Injunctive personal norms as a DV	c’ model	0.02 (0.01)	2.32 *	0.01, 0.06	0.02 (0.01)	2.32 *	0.01, 0.06
	a model	0.03 (0.01)	2.12 *	0.01, 0.07	0.03 (0.01)	2.12 *	0.01, 0.07
	b model	0.02 (0.01)	2.32 *	0.01, 0.06	0.02 (0.01)	2.32 *	0.01, 0.06
Behavioral outcome as a DV	c’ model	0.06 (0.03)	2.06 *	0.02, 0.15	0.02 (0.01)	1.94 †	0.01, 0.06
	a model	0.05 (0.02)	2.26 *	0.02, 0.11	0.02 (0.01)	2.14 *	0.01, 0.05
	b model	0.06 (0.03)	2.06 *	0.02, 0.15	0.02 (0.01)	1.93 †	0.01, 0.06

c’ path, injunctive societal norms → behavioral outcome; a, injunctive societal norms → injunctive personal norms; b, injunctive personal norms → behavioral outcome; ^†^*p* < 0.10, *p < 0.05, **p < 0.01, ***p < 0.001.

The conditional effect of IDV on a-path was significant, such that IDV positively moderated the relationship between injunctive societal norms and injunctive personal norms. As the individualism score increased from country to country, the influence of societal on personal norms also increased (wth coeff. = 0.002, LL/UL = 0.001–0.002, Figures 3, 5). No additional significant conditional effects were found in the model with IDV as a moderator and CPI as a DV (Figure 3, significant path moderation is highlighted by blue arrows). H5 was partially supported by indicated differences by IDV in a-path.

The conditional effects of IDV on paths a, b, and c’ were significant in the model with this cultural dimension as a moderator and NDCP as a DV. The interaction effects of IDV and (a) injunctive societal norms and (b) injunctive personal norms on NDCP were negative. In more individualistic countries, higher perceptions of injunctive norms, both societal (wth coeff. = −0.001, LL/UL = –0.001 – –0.0003) and personal (wth coeff. = −0.001, LL/UL = –0.0017 – –0.0007), were associated with lower instances of NDCP compared to more collectivistic countries (Figure 5, significant path moderation is highlighted by blue arrows). H6 was supported with IDV moderating paths a, b, and c’ with NDCP as a DV.

No direct between-group effects of IDV on injunctive personal norms and CPI and NDCP were significant. Additional mixed-models analysis also indicated no significant effects of IDV on injunctive societal norms.

### 4.4 PD and IDV interaction relationships

**Figure 6:**
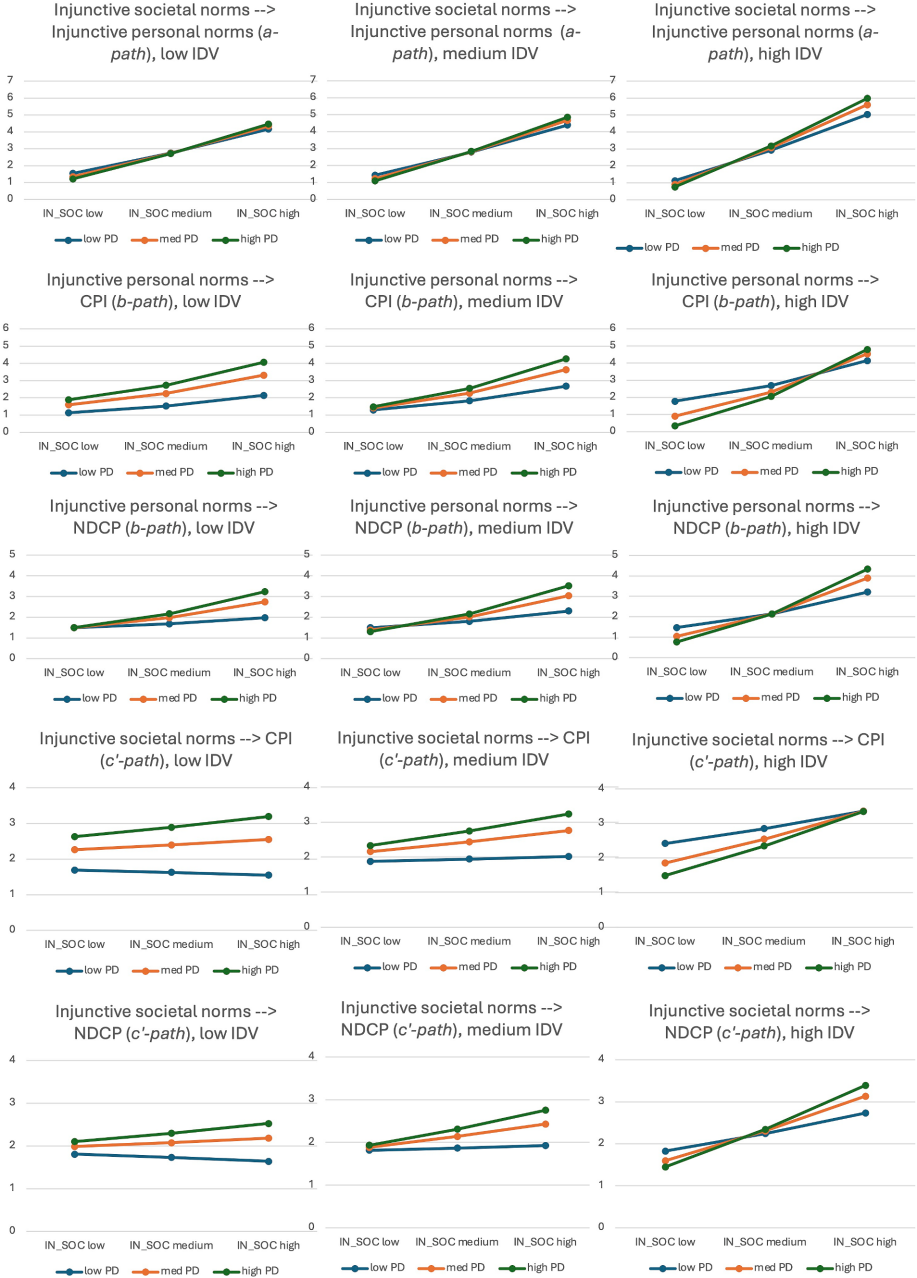
Three-way interactions of individualism/collectivism, power distance, and injunctive norms on counterfeit purchase intentions and non-deceptive counterfeit purchase.

The interaction between PD and IDV did not significantly predict the relationships among injunctive societal and personal norms and each of the two DVs. Significant within-group three-way interactions of PD, IDV, and injunctive social norms on both outcome variables were significant (all *p*-values <0.001). Figure 6 demonstrates the differences in associations between injunctive societal and personal norms (a-path), injunctive personal norms and CPI/NDCP (b-path), and injunctive societal norms and CPI/NDCP (c’-path). In summary, higher levels of IDV and PD were associated with a stronger relationship between societal and personal injunctive norms. With the increase of IDV, the differences in CPI across PD levels decreased when injunctive personal norms perceptions of counterfeit buying were high. These differences were more pronounced in low and medium IDV countries. Similarly, more pronounced associations were found in high IDV countries with injunctive social norms as an IV. Finally, it is at the level of high IDV that the differences among the levels of PD were more pronounced in moderating the relationships between injunctive norms (both personal and societal) and NDCP.

## 5 Discussion

Using the hierarchical norms (Patrick et al., 2012) and cultural dimensions theoretical frameworks (Hofstede, 1979; Hofstede and Bond, 1984; Hofstede et al., 2010), the present 17-country study focused on individual- and country-level predictors of counterfeit purchase intentions and past behaviors. Two types of injunctive norms, societal and personal, were used as individual-level serial predictors of counterfeit-related outcomes, where respondents’ perceptions of counterfeit buying approval were measured in distant and close social circles. We also proposed that country-specific characteristics, such as cultural dimensions, would predict norms and counterfeit-related variables and moderate their relationships. We focused on two cultural dimensions of individualism/collectivism and power distance, which we theorized would be most relevant to the social norms approach.

### 5.1 Summary of findings

Study analyses consistently indicated the significance of injunctive personal norms that positively mediated the relationships between injunctive societal norms and counterfeit purchase and intentions. This relationship emerged both at the individual and country level. Thinking that buying counterfeits is accepted by distant peers (societal) strengthened the perceptions that this behavior is approved by proximal peers (personal), which increased the expression of CPI and was associated with a higher frequency of buying counterfeits. Moreover, this mediation relationship held significance at the country level, where country averages for the outcome variables were higher through the effect of country-averaged injunctive societal norms mediated by injunctive personal norms. The results suggest that injunctive *personal* norms positively predict counterfeit buying behavioral outcomes in this study. When analyzing the direct relationship between injunctive societal norms and the outcome variables, the within-group effects were positive and significant; yet they were weaker than the indirect relationships and the relationships of injunctive personal norms. An interesting finding of the study was that the between-group effects of injunctive societal norms on counterfeit behavioral outcomes were either negative or non-significant. This means that greater perceptions of social approval of counterfeit buying, averaged for each country sample, were associated with lower levels of counterfeit behaviors and intentions.

Our findings generally supported past evidence (e.g., Patrick et al., 2012; Alhabash et al., 2021), though the context is different. Both Patrick et al. (2012) and Alhabash et al. (2021) examined risky alcohol use, whereas the current study looked at buying counterfeits. These scholars also did not focus on injunctive norms that was central to our study. The extension of the hierarchical norms approach to studying counterfeit buying behavior further supports the importance of, first, examining normative perceptions at various reference group levels, and, second, understanding how personal norms, though they directly predict behavior, are strengthened when they are aligned with societal norms.

Although cultural dimensions did not predict injunctive societal and personal norms or counterfeit-related behaviors and intentions, both PD and IDV moderated the injunctive societal-personal norms relationship. The moderation was positive for IDV and negative for PD. The influence of injunctive societal norms on injunctive personal norms was stronger in individualistic than collectivistic countries. This relationship was the opposite when moderated by PD. The association between societal and personal injunctive norms was weaker in higher than lower PD countries. In other words, in individualistic countries and countries with low power distance index, perceptions of social approval of counterfeit buying worked in conjunction with the beliefs about close friends and families approving of such behaviors. This interesting finding adds to the body of empirical evidence that suggests mixed relationships between culture and counterfeit-related phenomena (e.g., Santos and Ribeiro, 2006).

IDV negatively moderated the relationship between injunctive personal norms and knowingly buying counterfeits (NDCP). In collectivistic countries, the beliefs about close friends’ and family’s acceptance of counterfeit buying were strongly associated with past purchase of counterfeit products. In individualistic countries, such beliefs have a weaker influence on this behavior. Furthermore, in countries with high PD, injunctive personal norms led to a greater increase in NDCP and CPI than in countries with low PD scores. This suggests that injunctive personal norms, or the perceptions of proximal social circles accepting counterfeit purchasing, play a much greater role in predicting counterfeit purchase behaviors than injunctive societal norms. In general, this result echoes the existing evidence related to cultural differences, social norms, and counterfeit attitudes and behavior (Ronkainen and Guerrero-Cusumano, 2001; Phau and Teah, 2009; Faria, 2013; Swoboda et al., 2014; Sharma et al., 2022). Our study also suggests a larger disconnect between societal and personal injunctive norms in collectivistic and high PD countries than in individualistic and low PD countries.

Moderation effects of the two cultural dimensions on the relationship between injunctive societal norms and the DVs indicated that in individualistic and low PD countries, the relationship between injunctive societal norms and NDCP was weaker than in collectivist and high PD countries. In other words, in collectivistic and high PD nations, consumer perceptions of counterfeit buying acceptance by distant others led to greater past purchases of counterfeits. This relationship was significant only for NDCP as the DV. It is plausible that in low PD nations, consumers attribute their actions to individual values rather than conformity with the social norms, whereas in high PD countries, consumers are more likely to explain buying counterfeits as an expression of being part of a national orientation that is more accepting of buying counterfeits. Furthermore, three-way interactions of IDV, PD, and injunctive norms on the outcome variables showed that in more individualistic countries that scored high on power distance, the relationships between injunctive societal norms and past counterfeit buying were the strongest. This finding calls for further investigation.

### 5.2 Theoretical and practical implications

The theoretical implications of the present study are related to the complex relationships among culture, injunctive societal and personal norms, and counterfeit buying phenomena. Our findings indicate that personal norms play a stronger role in predicting counterfeit purchase intention and behavior than societal norms. As expected, this relationship is stronger in collectivistic and high PD countries than in individualistic and low PD societies. Societal norms also have a stronger direct impact on buying counterfeits knowingly in collectivistic and high PD than individualistic and low PD countries.

The study results indicated that injunctive societal norms had a stronger associations with injunctive personal norms in individualistic and low PD countries than in collectivistic and high PD nations. This finding changes the prior perceptions of the linear and positive relationships between collectivism and the influence of social norms. Perhaps the disconnect between societal and personal norms is larger in collectivistic and high PD societies, with the perceptions of close friends and family as having a much stronger influence on consumer decisions and intentions than abstract distant others. At the same time, societal and personal norms seem to be working in unison in individualistic and low PD countries where the influence of personal norms on consumer behaviors is weaker.

Taken together, our findings offer a set of practical implications for developing awareness and behavior change interventions and campaigns tailored to different countries based on their cultural orientations. Adjusting normative misperceptions about proximal social circles in collectivistic countries could be beneficial to changing consumers’ attitudes and behaviors toward buying counterfeit products. Highlighting the misperceptions related to the acceptance of buying counterfeits among close social groups could lead consumers to reconsider buying counterfeits as it pertains to showcasing the harmony between oneself, embedded into a close social setting that is also harmonious with the larger national group. On the other hand, it is beneficial to pay equal attention to distant and proximal social relationships when applying the social norms approach (Berkowitz, 2005) to individualistic countries. Based on the study findings, in communities that are more collectivistic, messages that amplify social disapproval of buying counterfeits among closer friends and family might alter individual expectations more than depicting the disapproval of distant others, thus influencing behavioral compliance of refraining from buying counterfeits. In more individualistic societies, messages can embed references to distant and proximal social circles. For example, a public service announcement (PSA) targeting collectivistic communities could be set in a friends-and-family setting, stressing respect for social relations and familial values. A PSA targeting individualistic countries could depict multiple groups – from close to distant others – and connections among them to emphasize behavior disapproval at different social levels. Furthermore, message strategies should vary on the basis of power distance, where they do not always work in unison with individualism–collectivism. For example, messages emphasizing social status when buying genuine products can be most effective in individualist countries with high power distance scores.

### 5.3 Limitations and future research directions

A few limitations are worth noting. First, despite the fact that our quota-based sampling aimed at recruiting representative samples from the 17 countries, ours was a convenience sample, thus generalizability to the entire population within each country is limited. Future research should seek to collect data from randomly selected samples. At the same time, the convenience sample allowed us to recruit demographically similar groups of consumers that could explain low between-group variability. Second, our survey was cross-sectional, which limits causality inferences assumed when conducting mediation analyses. Given that our theoretical model replicates prior work on hierarchical norms and is based on interpretations of atemporal nature of cross-sectional variables (see Winer et al., 2016), we were able to implement mediation analysis as a statistical technique to estimate the hierarchical structure of social norms. Additionally, the very nature of the two levels of reference groups (distal vs. proximal) implies that close friend norms are embedded within societal norms. However, it is important to note the limitation of cross-sectional data in interpreting the results of the mediation analysis. Future research should further examine whether hierarchical norms are ordered temporally or atemporally. Third, our study did not measure power distance and individualism–collectivism at the individual level and rather leveraged country-level data that were included as a second-level variable in our multilevel analysis. Future research should strive to measure cultural values at the individual country levels. Finally, it is important to highlight that cultural values, as country-level variables, are averages of the entire population, thus the interpretation of our findings, both theoretically and practically, should consider within-country variability on these cultural values, hence the need for individual-level measurement of cultural values.

## 6 Conclusion

The current study showed that cultural differences are associated with normative influences on individual behavior. As individuals in collectivistic and high power distance cultures value social connections and respect social hierarchy, perceiving the act of buying counterfeits as acceptable at proximal levels facilitated enacting risky behaviors of buying counterfeits. On the other hand, individualism and low power distance facilitated stronger connections between normative perceptions of both distal and proximal.

## Data availability statement

The raw data supporting the conclusions of this article will be made available by the authors, without undue reservation.

## Ethics statement

The studies involving humans were approved by Michigan State University Institutional Review Board. The studies were conducted in accordance with the local legislation and institutional requirements. The participants provided their written informed consent to participate in this study.

## Author contributions

AK: Conceptualization, Formal analysis, Methodology, Writing – original draft, Writing – review & editing. PH: Conceptualization, Methodology, Writing – original draft, Writing – review & editing. MM: Writing – original draft, Writing – review & editing. HL: Writing – original draft, Writing – review & editing. SA: Conceptualization, Methodology, Project administration, Resources, Supervision, Writing – original draft, Writing – review & editing.

## Appendix 1

TABLE A1 Latent variable* Model fit statistics based on multi-group confirmatory factor analysis.

**Table:**

	**Overall fit**	**Configural fit**	**Metric fit**	**Scalar fit**
**CFI**	0.996	0.993	0.991	0.984
**TLI**	0.994	0.989	0.989	0.984
**RMSEA**	0.040	0.054	0.054	0.067
**SRMR**	0.010	0.016	0.027	0.034

Overall fit, unconstrained model; Configural fit, Country groups specified; Metric fit, factor loadings constrained, country group specified; Scalar fit, factor loadings and intercepts constrained, country group specified. *Three latent variables included in the path model: Injunctive Societal Norms, Injunctive Personal Norms, and Non-Deceptive Counterfeit Purchase.

## Appendix 2

TABLE A2 Factor and reliability analysis results by country.

**Table:**

	**Injunctive Societal Norms**			**Injunctive Personal Norms**			**Counterfeit Purchase Intentions (CPI)**		
	**EV**	**% of VE**	**α**	**EV**	**% of VE**	**α**	**EV**	**% of VE**	**α**
Argentina	2.56	85.22%	0.913	2.74	91.46%	0.953	2.49	83.03%	0.896
Australia	2.82	94.14%	0.969	2.84	94.78%	0.972	2.68	89.45%	0.941
Brazil	2.54	84.55%	0.909	2.71	90.22%	0.946	2.56	85.27%	0.912
Canada	2.63	87.71%	0.93	2.71	90.45%	0.947	2.46	81.84%	0.888
China	2.65	88.38%	0.934	2.67	89.12%	0.939	2.5	83.15%	0.899
Egypt	2.68	89.47%	0.941	2.71	90.45%	0.947	2.73	90.84%	0.95
India	2.65	88.17%	0.933	2.71	90.38%	0.947	2.75	91.70%	0.954
Italy	2.57	85.59%	0.916	2.75	91.71%	0.955	2.72	90.57%	0.948
Kenya	2.37	78.90%	0.866	2.57	85.53%	0.915	2.08	69.36%	0.778
Mexico	2.59	86.25%	0.92	2.78	92.77%	0.961	2.65	88.46%	0.934
Nigeria	2.44	81.16%	0.884	2.69	89.68%	0.942	2.15	71.63%	0.8
Peru	2.57	85.74%	0.917	2.75	91.73%	0.955	2.49	82.89%	0.892
South Korea	2.53	84.45%	0.907	2.61	86.98%	0.924	2.65	88.26%	0.933
Spain	2.69	89.76%	0.943	2.79	91.13%	0.963	2.76	92.14%	0.957
United Arab Emirates	2.63	87.68%	0.93	2.7	90.08%	0.945	2.66	88.55%	0.935
United Kingdom	2.75	91.72%	0.955	2.8	93.37%	0.964	2.56	85.26%	0.913
United States	2.77	92.29%	0.958	2.84	94.63%	0.972	2.72	90.51%	0.947

EV, eigenvalue; % of VE, % of Variance Explained; α, Cronbach’s α.
